# Resource endowment, medical services utilization and health poverty vulnerability: evidence from middle-aged and older households in rural western China

**DOI:** 10.3389/fpubh.2026.1793479

**Published:** 2026-05-14

**Authors:** Jiahui He, Wenlong Wang, Ximin Ma, Qi Hu, Hui Qiao

**Affiliations:** 1 School of Public Health, Ningxia Medical University, Yinchuan, Ningxia, China; 2 Key Laboratory of Environmental Factors and Chronic Disease Control, Yinchuan, Ningxia, China; 3 School of Management, Shandong Second Medical University, Weifang, Shandong, China

**Keywords:** Bourdieu’s Capital Theory, health poverty vulnerability, medical services utilization, quantile regression, structural equation models

## Abstract

Health poverty vulnerability is a potential internal cause of poverty and return to poverty due to illness. Middle-aged and older households are risk groups. This study uses quantile regression and structural equation model to investigate the resource endowment and medical services utilization on health poverty vulnerability among middle-aged and older households. Results show: The number of the rural middle-aged and older households falling into poverty due to health factors is 2,121, accounting for 48.36%. The resource endowment and medical services utilization have a greater impact on middle-aged and older rural households with moderate vulnerability. Physical capital has the greatest impact on health poverty vulnerability. This study analyses the health poverty vulnerability of middle-aged and older households in rural western China from a dual perspective, in order to identify vulnerable groups and strengthening dynamic monitoring.

## 1 Introduction

### 1.1 From static poverty alleviation to dynamic risk prevention

Poverty reduction remains one of the core Sustainable Development Goals for the global community. While World Bank data shows a sustained decline in the global incidence of extreme poverty, poverty is inherently a dynamic rather than static state (1, 2). In 2020, China officially eliminated absolute poverty nationwide, but households still face persistent risks of falling into or returning to poverty due to unexpected shocks, especially health-related shocks (3). This reality has driven a global paradigm shift in poverty governance. From ex-post alleviation of existing poverty to ex-ante prevention of future poverty risk, with poverty vulnerability emerging as the core analytical tool for this forward-looking governance framework (4, 5).

### 1.2 Poverty vs. poverty vulnerability

To clarify the core analytical scope of this study, we first systematically distinguish between poverty and poverty vulnerability with critical reference to classical theoretical frameworks. Poverty is an ex-post, static measure of a household’s current welfare state, defined as income or consumption falling below a specified poverty line (6). It reflects a household’s actual deprivation at a given point in time, but cannot predict its future risk of falling into poverty. In contrast, poverty vulnerability was first formally defined by the World Bank as the probability that a household will fall below the poverty line in the future due to exposure to adverse shocks (4). It is an ex-ante, dynamic predictive indicator that focuses on future risk rather than current deprivation, enabling the identification of at-risk groups before they experience poverty (5). Critically, existing research has confirmed that traditional poverty-targeted policies often fail to cover households that are not currently poor but have high vulnerability, which are the main sources of new and returning poverty (7, 8). This critical distinction highlights the necessity of using vulnerability as the core outcome variable in this study, rather than just current poverty status, to provide a scientific basis for pre-emptive anti-poverty policies.

### 1.3 Health poverty vulnerability

Illness is the leading cause of poverty and return to poverty in China, accounting for over 40% of registered poor households annually (9, 10). Against this backdrop, health poverty vulnerability has become the core focus of research on health-related poverty. In this study, we provide a clear and concise definition. Health poverty vulnerability is the probability that a household’s welfare level will fall below the relative poverty line in the future, specifically driven by health shocks. We further clarify the conceptual boundary of health poverty vulnerability by distinguishing it from two related but distinct concepts. Unlike general poverty vulnerability, which covers risks from all economic, environmental and social shocks, health poverty vulnerability focuses exclusively on the transmission mechanism of health-induced shocks, with a more targeted scope for analyzing illness-related poverty (11). Catastrophic health expenditure (CHE) defined as out-of-pocket medical expenditure exceeding 40% of a household’s non-subsistence expenditure (9), is a key driver of health poverty vulnerability but not equivalent to it. CHE is an ex-post measure of current medical burden, while health poverty vulnerability captures the long-term, dynamic risk of persistent poverty resulting from the interaction between health shocks and household resource scarcity, including two core pathways: cost-driven poverty (catastrophic medical expenditure) and income-loss poverty (diminished labor capacity due to ill health) (12).

In this study, health poverty vulnerability is operationalized as the estimated probability of a household falling below the 60% relative poverty line due to health shocks, with a vulnerability threshold of 0.5 (i.e., households with a probability ≥50% are classified as high vulnerability), consistent with mainstream international practices (5, 12).

### 1.4 Resource endowment, medical services utilization and health poverty vulnerability

This study builds on Bourdieu’s Capital Theory and Andersen Behavioral Model of Health Services Use to elaborate the logical linkage between our key variables. Based on Bourdieu’s Capital Theory, we conceptualize household resource endowment as three interrelated dimensions: human capital, physical capital and social capital (13, 14). Resource endowment is the core foundation for households to cope with health shocks. Households with richer resource endowments have stronger capacity to buffer the economic impact of illness, and thus lower health poverty vulnerability (15). Specifically, physical capital provides direct material protection against medical expenditure shocks. Human capital affects households’ health literacy, labor capacity and long-term income-generating ability. Social capital provides informal insurance through social networks and reciprocal support (16, 17). Based on the Andersen Behavioral Model, we articulate the mediating role of medical service utilization in the relationship between resource endowment and health poverty vulnerability (18). The model identifies resource endowment as a core “enabling factor” that determines whether households can translate health needs into effective, timely medical service utilization. Households with richer resource endowments are more likely to use outpatient, inpatient and preventive health services, which can prevent minor illnesses from developing into serious chronic conditions, reduce long-term medical expenditure, and avoid permanent labor capacity loss, thereby lowering health poverty vulnerability (19, 20).

However, existing research has three key gaps: (1) Most studies focus on the separate impact of resource endowment or medical service utilization on health poverty vulnerability, with few examining their joint impact and internal transmission mechanism; (2) Existing research mostly uses mean regression to analyze the average effect, ignoring the heterogeneous impact on households with different vulnerability levels; (3) Few studies have targeted middle-aged and older households in western rural China, the highest-risk group for illness-related poverty, leading to a lack of targeted empirical evidence for this group. This study precisely targets these three gaps to provide new evidence for preventing health poverty in western rural China.

### 1.5 Research context and subject rationale

This study focuses on middle-aged and older rural households in Ningxia, western China. We define our study subjects as households with a household head aged 45 years or older, which is supported by strong theoretical and empirical evidence.

First, theoretical support from life-cycle theory and health capital depreciation theory. According to the life-cycle theory and Grossman’s health capital model, age 45 is a critical turning point for rural residents engaged in long-term manual labor (21, 22). After 45, physical function declines rapidly, health capital depreciates significantly, and the ability to resist health shocks weakens sharply, which directly increases the risk of health poverty vulnerability (22). Second, empirical evidence from mainstream national surveys. The China Health and Retirement Longitudinal Study (CHARLS) is the most authoritative national survey for middle-aged and older Chinese. It also uses 45 years old as the starting age for investigating health, labor, and economic vulnerability (23). This criterion has been widely adopted in studies on health poverty and vulnerability in rural China (12, 19). Third, empirical evidence specific to rural western China. In western rural areas dominated by heavy physical farming, residents aged 45 + have a much higher prevalence of chronic diseases, more frequent health shocks, and lower labor capacity than younger adults (24, 25). Studies confirm that this group bears the highest medical cost burden and faces the greatest risk of illness induced poverty (8, 26). Fourth, structural vulnerability of rural households. Middle-aged people (45–59) are often the main labor force and income earners in rural households. Once they suffer health shocks, the whole household easily falls into poverty (12). Meanwhile, this group is transitioning into old age, with insufficient pension security and limited access to medical services, making them the core high-risk group for health poverty vulnerability that must be focused on (19, 27).

### 1.6 Study design and marginal contributions

Using 2022 survey data from 4,371 middle-aged and older rural households in 171 villages of 4 counties in southern Ningxia, this study empirically examines the impact and transmission mechanism of resource endowment and medical service utilization on health poverty vulnerability. Our marginal contributions are as follows: First, we construct a household health poverty vulnerability evaluation index system adapted to the context of western rural China, and measure health poverty vulnerability using the Vulnerability as Expected Poverty (VEP) framework with a three-stage Feasible Generalized Least Squares (FGLS) model, providing a scientific measurement tool for identifying at-risk households. Second, we use quantile regression to explore the heterogeneous impact of resource endowment and medical service utilization on households with low, moderate and high health poverty vulnerability, identifying the most sensitive groups for targeted policy intervention. Third, we build an integrated theoretical framework from the dual perspective of resource endowment and medical care, and use structural equation modeling (SEM) to test the mediating role of medical service utilization, clarifying the internal mechanism between the core variables.

The remainder of this paper is organized as follows: Section 2 presents the theoretical analysis and research hypotheses; Section 3 describes the data, variables and methods; Section 4 reports the empirical results; Section 5 discusses the findings; Section 6 presents the limitations and future research directions; Section 7 concludes the study and proposes policy recommendations.

## 2 Theoretical analysis and frameworks

### 2.1 Bourdieu’s Capital Theory

In the specific context of rural western China, health poverty vulnerability is not merely a static deficiency of assets but a dynamic failure of “capital reproduction” where health shocks deplete human capital, which in turn hinders the accumulation of physical and social capitals. Bourdieu’s theory is more adaptable here as it treats social capital and human capital as mutually reinforcing powers that determine a household’s position in the health poverty field, providing a more rigorous basis for the structural equation modeling used in this research. Furthermore, this study deliberately employs Bourdieu’s theory of capital rather than the general framework for sustainable livelihoods because Bourdieu’s theory emphasizes how human capital, physical capital, and social capital are structurally “transformed” into defensive resources within the health sector. Unlike the general framework, Bourdieu’s theory allows us to operationalize “resource endowments” as a series of power relations, in which capital can mitigate health shocks through direct economic protection and indirect social reciprocity.

It is highly adapted to the livelihood characteristics of our study population. Our study focuses on middle-aged and older (45+) rural households in western China, who rely heavily on manual labor and face accelerated health capital depreciation. The sustainable livelihoods includes natural capital as a core dimension, but for this study population, cultivated land has extremely low liquidity and cannot be quickly converted to cope with sudden health shocks (25). In contrast, Bourdieu’s three-dimensional capital framework (human, physical, social) focuses on convertible capital that can be mobilized to buffer health shocks, which is more consistent with the actual livelihood strategies of rural households facing illness risks. For the health needs of middle-aged and older individuals, it is essential to receive timely medical treatment when ill, utilize medical services effectively, and not delay treatment due to financial constraints. The family serves as the primary guardian and should possess sufficient resource endowment to cope with crises. Scholars describe resource endowment through human capital, physical capital, and social capital (28).

Therefore, this study incorporates medical services utilization, combining human capital, physical capital, and social capital to investigate the impact on health poverty vulnerability in rural households of western China. Under the framework of Bourdieu’s Capital Theory, life-cycle theory and health capital theory, the age of 45 represents the stage where human capital, specifically health and physical strength begin to depreciate rapidly in rural settings (21, 22). While domestic standards often distinguish between middle age (45–59 years) and old age (60 years and above), our study aggregates these groups to analyze the continuous accumulation of health risks and their subsequent impact on household economic stability. Empirical studies have confirmed that the 45 + group in rural China has 2–3 times higher health poverty vulnerability than those under 45 (12, 23). This approach allows for a holistic view of how health shocks affect the “prime” labor force of rural households as they transition into later life, providing a more comprehensive early-warning mechanism for health related poverty.

### 2.2 Auxiliary supporting theories and theoretical integration

Grossman’s Health Capital Model regards health as a core component of human capital: health capital depreciation accelerates with age, and health damage will directly reduce households’ labor capacity and income-generating ability, increasing the risk of poverty (21). Combined with Life-Cycle Theory, age 45 is a critical turning point for rural residents engaged in long-term heavy manual labor. After 45, health capital depreciates rapidly, chronic disease prevalence rises sharply, and the ability to resist health shocks weakens significantly (22). This provides a rigorous theoretical basis for our focus on households with a head aged 45+, and also supports the operationalization of the human capital dimension.

Andersen’s Behavioral Model identifies household resource endowment as the core enabling factor that determines whether households can translate health needs into effective, timely medical service utilization (18). This theory builds the logical bridge between the core independent variable (resource endowment) and the mediating variable (medical service utilization). Households with richer capital endowment have stronger enabling conditions to use outpatient, inpatient and preventive health services, which in turn affects their health poverty vulnerability. This provides a direct theoretical basis for the mediating mechanism analysis of this study.

Bourdieu’s Capital Theory defines the core dimensions of household resource endowment. Health Capital and Life-Cycle Theory explains the unique vulnerability of the 45 + rural group. Andersen’s Model clarifies the mediating role of medical service utilization. All theories jointly explain the formation mechanism of health poverty vulnerability in the study population.

### 2.3 Human capital

Human capital is an indicator for evaluating the knowledge, skills, and health status of households (17). According to relevant economic theories, poverty is attributed to a lack of human capital. Sen argues that individuals with lower human capital, such as limited education and poor health, are more likely to fall into poverty due to their diminished capacity to earn income (6). Guo observed that rural labour migration has become one of the ways to alleviate rural poverty (29). For middle-aged and older households, healthcare decisions and expenditures are influenced accordingly by the constraints of low human capital in the household (30). The abundance of human capital impacts the socio-economic capacity and health sensitivity to health of the entire household, thereby determining the extent of medical service utilisation and the household’s risk of poverty due to health shocks. Therefore, this study will examine human capital through indicators such as educational level, health status, household labor force size, and the number of migrant workers.

### 2.4 Physical capital

Physical encompasses the infrastructure and means of production that support farmers’ livelihoods, and serves as a crucial foundation for addressing unexpected risks and challenges. When exposed to risky shocks, reservable physical capital can be quickly mobilized, helping to buffer sudden household consumption and expenditures. As a result, the abundance of material assets influences the household’s poverty status (31). In studies of household poverty vulnerability, Chinese researchers have measured household physical capital using indicators such as the quantity of agricultural machines, durable products, building material, and the type of drinking water (25). Researchers have also used housing safety and drinking water types as indicators of physical capital in studies of poverty alleviation livelihood strategies (17). This study referred to previous literature and combined with the living characteristics of local rural households, selecting physical capital indicators such as productive agricultural machines, durable products, building materials, drinking water type, latrines type, cultivated area, and separation of housing and kitchen.

### 2.5 Social capital

Social capital was first proposed by Hanifan, who described it as “the relationships of goodwill, friendship, empathy and social interaction in the groups and families that make up a rural community” (32). Since then, social capital has garnered research attention in various fields, including public health. As an informal institution, social capital provides access to both tangible and intangible resources through social interaction and connections with others, namely social relationship networks. Some researchers have listed social capital as the third type of capital, following human and physical capital (33). The reciprocal effect of social capital on human relationships facilitates information sharing and can also provide emotional and practical support. To some extent, it can alleviate the limitations imposed by insufficient human and material capital, enhancing a family’s ability to cope with risks. The greater the social capital, the more favourable it is for farmers to access medical information and consolidate the effects of poverty eradication (16, 17). Therefore, in this study, cash gift is selected to represent the social capital of the household to describe the resource endowment along with human capital and physical capital. The selection of “cash gifts” as a proxy for social capital is based on the “Renqing” culture prevalent in rural western China. In these communities, gift exchange is the most visible and quantifiable measure of a household’s social network density and reciprocal obligations. High expenditure on cash gifts often signifies a broader social support network that can be activated as “informal insurance” during health emergencies. While this indicator may not capture the cognitive dimensions of social capital, its validity as a structural proxy for social resource access in rural China has been confirmed in previous large-scale household surveys (e.g., CFPS and CHARLS).

### 2.6 Medical services utilization

Previous results from the National Health Services Survey indicate that the medical service demand and expenditure of middle-aged and older people are significantly higher than the social average, which imposes a heavy financial burden on families and society. Due to the dual vulnerability in terms of health and society, the middle-aged and older population has become a central focus of the medical service system (19). Researchers have developed a method for distinguishing medical behavior decisions (whether or not to seek healthcare) and medical expenses (34). Household resource endowment can have multiple impacts on individual behavior and household behavior decisions, particularly on the medical services utilization for middle-aged and older people. Households with a large labour force, higher education levels and better economic conditions may be more inclined to use medical services. This study builds on existing researches and adopts four indicators: outpatient services utilization, inpatient services utilization, preventive services utilization, and self-treatment utilization.

**Figure 1:**
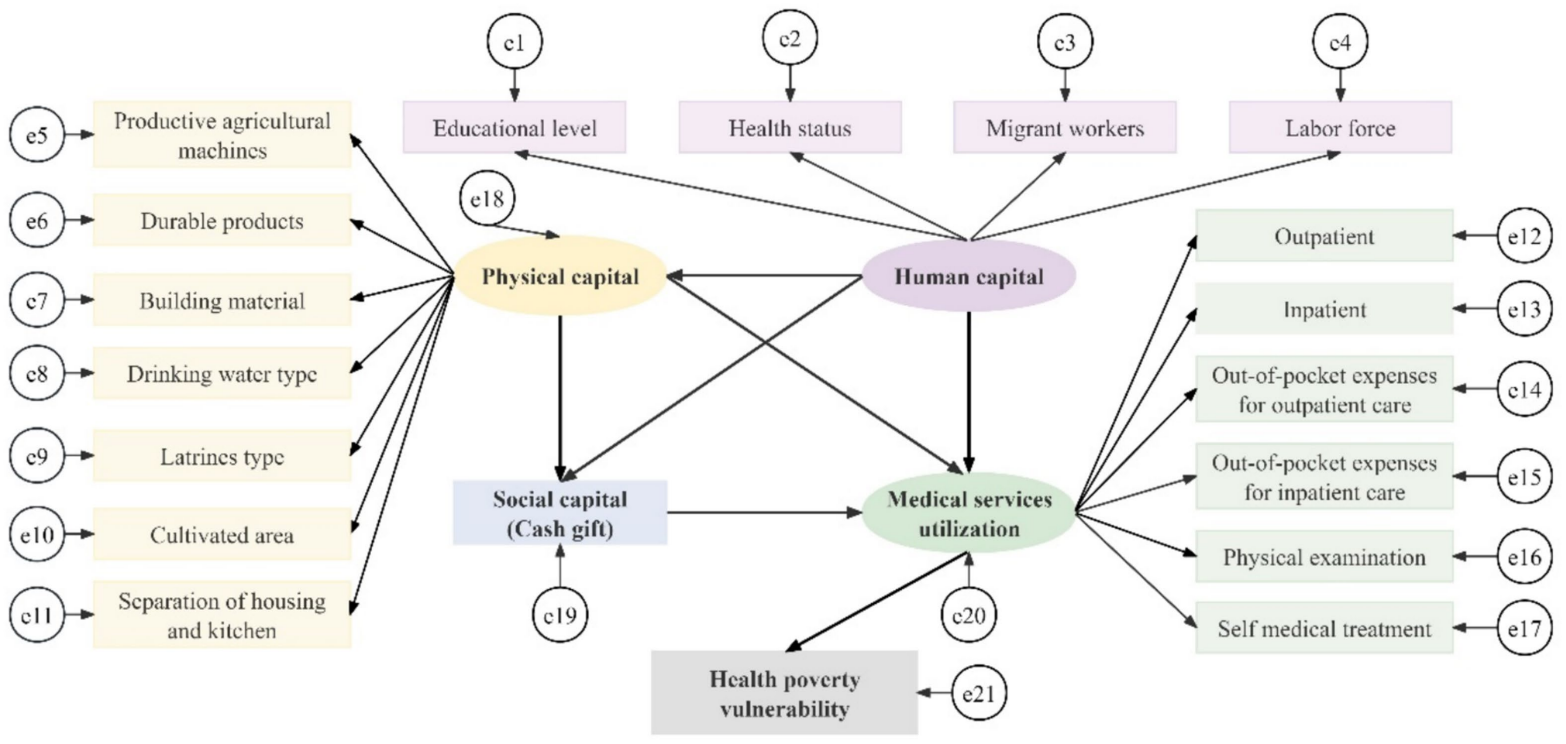
The initial model of resource endowment, medical services utilisation and health poverty vulnerability of middle-aged and older households in rural western China.

On the grounds of theoretical analysis and literature references, an initial model of the interaction path between resource endowment, medical services utilization, and health poverty vulnerability of rural households in western China is constructed, as shown in Figure 1.

### 2.7 The mediating mechanism of medical services utilization

The relationship between resource endowment and health poverty vulnerability is not only direct but also mediated by medical services utilization. According to the Andersen Behavioral Model of Health Services Use, resource endowments (such as human capital/health literacy and physical capital/wealth) serve as “enabling factors” that determine whether a household can translate health needs into actual medical utilization. Specifically, households with higher human capital are more likely to utilize preventive services, which reduces long-term vulnerability. Conversely, households with limited physical capital may delay treatment until it becomes an expensive inpatient emergency. Therefore, medical services utilization acts as a buffer mechanism: resource endowment improves the quality and timeliness of medical utilization, which in turn prevents the rapid depletion of household assets, thereby lowering health poverty vulnerability.

## 3 Methods

### 3.1 Data source

The data used in this study come from two logically linked sources, with a fully clarified 2009–2022 timeline. In 2009, Harvard University and Ningxia Medical University launched the collaborative pilot project Innovative Payment Systems to Enhance Healthcare Efficiency (further details available at: https://www.hsph.harvard.edu/re-alignment-health-system-incentives/sample-page/policy-impact-and-mediacoverage/). This project established a scientific, regionally representative sampling frame covering 4 counties of southern Ningxia, western China. The sampling frame adopted a standardized stratification criterion based on per capita disposable income, which has been verified by long-term research practice to have good stability and representativeness for rural populations in southern Ningxia. This study specifically uses the 2022 Rural Household Health Inquiry Survey data, funded by the National Natural Science Foundation of China and conducted based on the 2009 verified sampling frame. The survey systematically collected data on residents’ health status, healthcare service utilization, household resource endowment, and economic conditions, with the core aim of providing empirical evidence for preventing illness-induced poverty and return to poverty in western rural China. This study was conducted in full compliance with the Declaration of Helsinki. The study protocol was approved by the Ethics Committee of Ningxia Medical University (approval number: 2023-G157), and written informed consent was obtained from all participants prior to the survey.

### 3.2 Multi-stage stratified random sampling procedure

We adopted a three-stage stratified random sampling method for the 2022 survey, with a clear, replicable, and consistent procedure. We retained the 2009 project’s validated stratification criterion, and combined the 2021 per capita disposable income data to update the stratification of the 208 administrative villages in the sampling frame. All villages were divided into three strata: “good,” “medium,” and “poor” economic development levels, to ensure the sample covers the full economic heterogeneity of the study area. In each stratum, we used a simple random sampling method (random number table) to select the villages. A total of 171 administrative villages were finally included in the 2022 survey. The sampling process strictly followed the randomization principle. All villages in each stratum were numbered sequentially, and random numbers were generated to select sample villages, with duplicate or out-of-range numbers excluded. In each of the 171 sampled villages, we adopted a systematic sampling method to select 33 households from the full list of permanent resident households in the village. Permanent residents were defined as those who had lived in the village for ≥6 months in the past year. This definition ensures that the sampled households’ main health-seeking behavior, livelihood activities, and economic cycle are tied to the local area.

### 3.3 Sample size calculation and final sample screening

We calculated the minimum required sample size for this cross-sectional study using the standard sample size formula for estimating population prevalence in epidemiological and health economics research, with standardized formula and full calculation details as follows:


$$N=\frac{u_{\alpha }^{2}\pi (1-\pi )}{\delta^{2}}$$ (1)

In Equation 1, a two-tailed significance level of *α* = 0.05 was adopted, and the allowable error *δ* was set at 0.1π. According to existing literature, the incidence rate of health-related poverty vulnerability in rural areas of Ningxia was 28.15% (35), the minimum required sample size was calculated to be approximately 864 participants. The number of subjects ultimately included in this analysis exceeded this requirement, ensuring adequate statistical power.

We selected trained investigators for the survey, obtained informed consent from the survey subjects, and conducted a face-to-face questionnaire survey. After completing the questionnaire, the investigator will collect it on the spot and check for any incomplete or incorrect information. During the survey process, a daily review system was implemented, with a review team consisting of investigators, team leaders, and quality controllers checking the questionnaires at the end of each day to exclude those with missing or unclear values, ensuring the completeness and validity of the collected data. This study focused on collecting data related to basic information, medical services, health status, and household assets. Household-level data were integrated with individual-level data. Households were excluded if any household-level data required for the health poverty vulnerability measure were missing, or if individual-level data for all household members were incomplete. We screened the survey respondents for households where the head of the household was 45 years or older. After data collation, the final sample size consisted of 4,371 households and 15,108 individuals. The final valid sample size of this study is 4,371 households, which is more than 5 times the minimum required sample size. This not only fully meets the basic statistical requirements, but also ensures sufficient statistical power for subsequent heterogeneous analysis and structural equation modeling, avoiding the risk of type II error due to insufficient sample size.

### 3.4 Variables and definitions

Based on the theoretical analysis mentioned in the previous section, the appropriate variables were selected as indicators for the study. Resource endowment is characterized by three dimensions: human capital, physical capital, and social capital. Medical services utilization is divided into four dimensions: outpatient services utilization, inpatient services utilization, preventive services utilization and self-care utilization. The specific explanation of each variable is provided in Table 1. For continuous variables including cash gift and out-of-pocket expenses for outpatient care and out-of-pocket expenses for inpatient care, we take the natural logarithm to follow the standard econometric norm (36). This transformation can mitigate the right-skewed distribution characteristics of household economic variables, reduce the interference of extreme values on regression results, and alleviate potential heteroscedasticity, to ensure the unbiasedness and robustness of the estimation results.

**Table 1:** Explanation of the status quo of middle-aged and older households.

Variables			Explanation
Resource endowment	Human capital	Educational level	Household education level per adult (1 = no schooling, 2 = primary school, 3 = junior high school, 4 = high school and above)
		Health status	Chronic diseases or disability of household members or not (1 = yes, 0 = no)
		Labor force	Household labor force size
		Migrant workers	Number of household Migrant workers
	Physical capital	Productive agricultural machines	Number of household productive agricultural machines
		Durable products	Number of household durable products
		Building material	Household building material is kiln or not (1 = yes, 0 = no)
		Drinking water type	Household drinking water type is tap water or not (1 = yes, 0 = no)
		Latrines type	Household latrines type is flushing or not (1 = yes, 0 = no)
		Cultivated area	Square of cultivated area size in the household
		Separation of housing and kitchen	Separation of housing and kitchen or not (1 = yes, 0 = no)
	Social capital	Cash gift	Amount of gift expenses for household during the year
Medical services utilization	Outpatient services utilization	Outpatient	Utilization of outpatient services by family members within 2 weeks or not (1 = yes, 0 = no)
		Out-of-pocket expenses for outpatient care	Own expenses for outpatient service for household
	Inpatient services utilization	Inpatient	Utilization of inpatient services by family members during the year or not (1 = yes, 0 = no)
		Out-of-pocket expenses for inpatient care	Own expenses for inpatient service for household
	Preventive services utilization	Physical examination	Physical examination of family members within 1 year or not (1 = yes, 0 = no)
	Self-treatment utilization	Self medical treatment	Self-medication by family members within 2 weeks or not (1 = yes, 0 = no)

Specifically, *X_ht_* represents the factors affecting household income. In this study, *X_ht_* includes household demographics (size, dependency ratio), health status (chronic illness), and institutional protection (medical insurance type and medical debt). The inclusion of these health-related economic indicators minimizes estimation bias in the variance of future income, using 60% of the per capita disposable income (9,202 CNY). Considering that the research subject of this paper is rural areas in Western China, whose level of economic development and cost of living are highly comparable to those of low- and middle-income countries globally, the international poverty line of $3.65 per day, specifically used by the World Bank to monitor poverty in low- and middle-income countries, was selected. This standard can more accurately reflect the actual economic hardships faced by residents in this region. Following the criteria of Chaudhuri, a household is classified as having high health poverty vulnerability if its estimated probability (*V_ht_*) is ≥ 0.5, signifying a critical risk that warrants policy intervention in the context of China’s poverty alleviation monitoring (5).

### 3.5 Measurement of health poverty vulnerability

Researchers commonly use three measures of poverty vulnerability: vulnerability as expected poverty (VEP), vulnerability as expected utility (VEU), and vulnerability as uninsured expo-sure to risk (VER). Among these, the VEP is more widely used because it enables forward-looking poverty vulnerability results to be obtained using only cross-sectional data without the need of panel data (37). Notably, the core design purpose and academic consensus of the classic VEP framework is to use cross-sectional data to estimate the forward-looking probability of a household falling into poverty in the future, which has become the standard paradigm in global poverty vulnerability research, including health poverty vulnerability studies (5, 26). This design is fully aligned with the core objective of this study to assess the ex-ante risk of future health-induced poverty among rural households.

Since our study uses cross-sectional data, we apply the VEP to measure the vulnerability to health poverty of rural households, employing the three-stage Feasible Generalized Least Squares (FGLS) model (5, 38). The FGLS regression analysis results in this paper were obtained using Stata 17.0. The estimation method of vulnerability to relative health poverty is as follows:

Firstly, the future income equation is estimated and the residual square from the regression is used as the dependent variable for the ordinary least square (OLS) method.


$$\mathrm{Ln}Y_{\mathrm{ht}+1}=X_{\mathrm{ht}}\beta_{h}+e_{h}$$ (2)

$$\begin{array}{c} {\hat{e}}_{h}^{2}=X_{h}\theta +y_{h} \end{array}$$ (3)

Where $X_{\mathrm{ht}}$ in Equation 2 represents a set of variables characterizing the household characteristics, household health status and household economic status of disease. Considering the heterogeneity of the rural population in different counties, townships, and villages, in Equation 3, the residual square is considered as an approximation of the variance of income ${\hat{e}}_{h}^{2}$ , and the residual square from the regression are used as the dependent variable for OLS estimation.

Secondly, in Equation 4 and Equation 5, weighted regression of the logarithm of income and the residual square yield FGLS estimates $\hat{\beta }$ and $\hat{\theta }$ , which are used to estimate the expected value and variance of future income:


$$\begin{array}{c} \hat{E}[{\mathrm{LnY}}_{h}|X_{h}]=X_{h}\hat{\beta } \end{array}$$ (4)

$$\begin{array}{c} \hat{V}[{\mathrm{LnY}}_{h}|X_{h}]=X_{h}\hat{\theta } \end{array}$$ (5)

Finally, we assume that the logarithm of future income levels of rural households obeys normal distribution (39). This is the core standard assumption of the classic VEP framework, and is an inherent part of the method’s three-stage FGLS estimation design (5). The log-normal distribution of household income is also a long-standing academic consensus in development economics, systematically validated in the classic household survey methodology work, and has become the universal norm for poverty vulnerability research globally (40). Our specification of this assumption is fully consistent with the vast majority of authoritative studies on health poverty vulnerability in rural China, ensuring the validity of our measurement and the comparability of our results with existing literature (12, 26). Lnl in Equation 6 represents the logarithm of the relative poverty line. Considering that China has completely eliminated absolute poverty, relative income poverty will be used to measure income status. In accordance with international standards, we used the relative poverty line at 60% of per capita disposable income (26). Therefore, this study we chose to use 9,202 CNY/year calculated from the 2022 per capita disposable income of rural residents in Ningxia as the relative poverty line standard and used it as a key variable for subsequent research. Referring to the literature, we considered the likelihood of households falling into relative poverty in the future was higher than 50% as the vulnerability criterion (12). Then vulnerability to relative health poverty of rural households can be calculated:


$$\begin{array}{c} \mathrm{vu}l_{\mathrm{ht}}=\hat{P}(\mathrm{Ln}Y_{h}\leq \mathrm{Lnl}|X_{h})=\phi (\frac{\mathrm{Lnl}-X_{h}\hat{\beta }}{\sqrt{X_{h}\hat{\theta }}}) \end{array}$$ (6)

### 3.6 Statistical analyses

Epidata 3.1 was used to input the survey data, and Stata17.0 was used to describe the status quo of middle-aged and older rural households. Descriptive data is expressed as frequencies and mean ± standard deviation.

Since the OLS approach measures only the effect of explanatory variables on the mean expected value of the dependent variable, it is not possible to directly analyze how households resource endowment and medical services utilisation change with the health poverty vulnerability. Additionally, OLS assumes that the dependent variable follows a normal distribution. Therefore, this study used quantile regression, with resource endowment and medical services utilization as independent variables, and the health poverty vulnerability index (ranging from 0 to 1) as the dependent variable. Compared with OLS, quantile regression is more robust to outliers, normality of explained variables, or heteroscedasticity issues. Prior to data analysis, we performed the collinearity test. Following the quantile regression, we conducted robustness testing using the full quantile regression model. Throughout the analysis, we used Stata 17.0 for statistical analysis. *p* values less than 0.05 was considered statistically significant.

Quantile regression and structural equation modeling (SEM) were used to analyze the data. Quantile regression was employed to analyze the heterogeneous impact of resource endowment across the 25th, 50th, and 75th quantiles (representing low, moderate, and high vulnerability levels), while SEM was used to test the mediating paths of medical services utilization. This study employs a formative measurement model. Since the observed indicators are all objective measures rather than subjective perceptual scales, traditional internal consistency reliability tests (such as Cronbach’s alpha) are not applicable. Following the practices of existing research, this paper ensures measurement quality by verifying the content validity of the selected indicators through a literature review, which has been elaborated in detail in the theoretical analysis and framework. Model adjustments were made only when theoretically justifiable based on Modification Indices to avoid data-driven overfitting. Model construction and adjustment were operated in IBM SPSS Amos 28.0. Prior to analysis, data cleaning was performed to ensure data integrity. Cases with missing values on any of the key study variables were excluded, resulting in a complete dataset with no missing data for the structural equation modeling analysis.

## 4 Results

### 4.1 Description of the status quo of middle-aged and older households

A total of 4,371 households participated in the study. According to the corrected data in Table 2, The average educational level was 2.21 ± 0.75. 69.27% (3,028) of the householders reported poor health, while 30.73% (1,343) reported good health. Nearly all households had access to tap water (95.49%). The average cash gift (Logarithmization) was 6.44 ± 3.38. Furthermore, 12.29% of the households sought medical care, with an average outpatient own expense (Logarithmization) was 0.84 ± 2.32. The majority of households had undergone medical examination (71.22%).

**Table 2:** Description of the status quo of middle-aged and older households (*N* = 4,371).

Variables	Categories	Mean±SD	Frequency (N)	Percentage (%)
Human capital				
Educational level		2.21 ± 0.75		
Health status	Yes		3,028	69.27
	No		1,343	30.73
Labor force		2.39 ± 1.66		
Migrant workers		0.37 ± 0.75		
Physical capital				
Productive agricultural machines		0.42 ± 0.69		
Durable products		10.03 ± 3.22		
Building material	Yes		33	0.75
	No		4,338	99.25
Drinking water type	Yes		4,174	95.49
	No		197	4.51
Latrines type	Yes		387	8.85
	No		3,984	91.15
Cultivated area		22.79 ± 44.80		
Separation of housing and kitchen	Yes		3,277	74.97
	No		1,094	25.03
Social capital				
Cash gift (Logarithmization)		6.44 ± 3.38		
Outpatient services utilization				
Outpatient	Yes		537	12.29
	No		3,834	87.71
Out-of-pocket expenses for outpatient care (Logarithmization)		0.84 ± 2.32		
Inpatient services utilization				
Inpatient	Yes		1,570	35.92
	No		2,801	64.08
Out-of-pocket expenses for inpatient care (Logarithmization)		2.92 ± 3.99		
Preventive services utilization				
Physical examination	Yes		3,113	71.22
	No		1,258	28.78
Self-treatment utilization				
Self medical treatment	Yes		452	10.34
	No		3,919	89.66

### 4.2 The measurement analysis of health poverty vulnerability

This paper applied the VEP method in conjunction with the FGLS model to calculate the health poverty vulnerability of rural middle-aged and older households in Ningxia in 2022. The estimated results are presented in Table 3. With a relative poverty line set at 9,202 CNY/year and a vulnerability threshold of 0.5 for measuring health poverty vulnerability, 2,121 rural middle-aged and older households were found to fall into poverty due to health factors, accounting for 48.36%. The robustness test results show that 2,027 rural middle-aged and older households were found to fall into poverty due to health factors, accounting for 46.37%. The robustness tests confirmed that the core findings did not shift significantly.

**Table 3:** Distribution of relative health poverty vulnerability index.

Vulnerability index	The sample size (%)	Mean	Standard deviation
[0, 0.25)	1,746 (39.95)	0.050	0.070
[0.25, 0.50)	511 (11.69)	0.374	0.074
[0.50, 0.75)	634 (14.50)	0.636	0.072
[0.75, 1]	1,480 (33.86)	0.903	0.068

### 4.3 Quantile regression analysis

#### 4.3.1 Testing for collinearity

To ensure the validity of the estimation results, the Variance Inflation Factor (VIF) was adopted to test the collinearity of each variable before conducting model estimation. A VIF value greater than 10 indicates the presence of collinearity. The result shows that the maximum VIF value is less than 2.5, indicating that there is no multicollinearity among the explanatory variables. Therefore, the analyses are valid.

#### 4.3.2 Regression results

This paper based on OLS analysis to examine the impact of resource endowment and medical services utilization on the health poverty vulnerability. Commonly used percentiles (0.25, 0.50, and 0.75) are selected for comparative analysis to observe the differing effects of resource endowment and medical services utilization on the distribution of health poverty vulnerability. These three quantiles represent rural middle-aged and older households with low, moderate, and high health poverty vulnerability, respectively. The regression results are presented in Table 4, where the dependent variable is the health poverty vulnerability of rural middle-aged and older households.

**Table 4:** Quantile regression and OLS results of resource endowment, medical services utilization and health poverty vulnerability of middle-aged and older households.

Explanatory variables	OLS	Quantile		
		25th quantile	50th quantile	75th quantile
Educational level	−0.0783* (0.0089)	−0.0411* (0.0066)	−0.1353* (0.0173)	−0.0820* (0.0108)
Health status	0.0544* (0.0120)	0.0367* (0.0090)	0.0838* (0.0231)	0.0354* (0.0139)
Labor force	0.0573* (0.0046)	0.0356* (0.0042)	0.0892* (0.0072)	0.0500* (0.0052)
Migrant workers	0.0540* (0.0077)	0.0558* (0.0154)	0.0831* (0.0132)	0.0434* (0.0068)
Productive agricultural machinery	−0.0474* (0.0080)	−0.0257* (0.0062)	−0.0708* (0.0137)	−0.0415* (0.0147)
Durable products	−0.0127* (0.0020)	−0.0089* (0.0020)	−0.0191* (0.0035)	−0.0109* (0.0020)
Building materials	0.0398 (0.0613)	0.0439 (0.0879)	0.0582 (0.0891)	0.0066 (0.0765)
Drinking water type	−0.0400 (0.0257)	−0.0096 (0.0361)	−0.0649 (0.0405)	−0.0282 (0.0202)
Latrines type	−0.0427* (0.0188)	−0.0150 (0.0111)	−0.0637 (0.0341)	−0.0513* (0.0259)
Cultivated area	−0.0005* (0.0001)	−0.0006* (0.0002)	−0.0011* (0.0006)	−0.0010 (0.0006)
Separation of housing and kitchen	0.0060 (0.0124)	0.0015 (0.0106)	0.0038 (0.0239)	0.0068 (0.0117)
Cash gift	−0.0277* (0.0017)	−0.0243* (0.0035)	−0.0414* (0.0033)	−0.0215* (0.0017)
Outpatient	0.1062 (0.0663)	0.1027 (0.0706)	0.1652 (0.1029)	−0.0343 (0.0683)
Out-of-pocket expenses for outpatient care	−0.0215* (0.0094)	−0.0185* (0.0094)	−0.0326* (0.0156)	−0.0022 (0.0103)
Inpatient	0.2661* (0.0523)	0.1940* (0.0532)	0.3504* (0.0889)	0.1896* (0.0623)
Out-of-pocket expenses for inpatient care	−0.0373* (0.0063)	−0.0253* (0.0061)	−0.0500* (0.0104)	−0.0270* (0.0079)
Physical examination	0.0009 (0.0119)	0.0230* (0.0086)	0.0088 (0.0234)	−0.0164 (0.0123)
Self medical treatment	−0.0208 (0.0176)	−0.0037 (0.0132)	0.0056 (0.0349)	−0.0252 (0.0167)

Standard errors are in parentheses. Standard errors for quantile regression are estimated using bootstrap techniques with 1,000 replications.

*p<0.05.

Health status, labor force, migrant workers and inpatient have statistically significant positive effects in all quantiles of the distribution of health poverty vulnerability distribution of rural middle-aged and older households. The overall trend showed inverted a V-shaped relationship. In addition, educational level, household productive agricultural machines, durable products, cash gift and out-of-pocket expenses for inpatient care have statistically significant negative effects. The overall trend showed a V-shaped relationship. That is to say, compared to households with low and high health poverty vulnerability, the impact whether positive or negative of resource endowment and medical services utilization is more pronounced on middle-aged and older rural households with moderate vulnerability.

Table 5 reports the Wald test results for coefficient differences across quantiles of the explanatory variables. The joint Wald tests indicate that the coefficients of the following explanatory variables differ significantly across the 0.25 percentile, 0.50 percentile, and 0.75 percentile. Further pairwise tests show that for health status, labor force, and inpatient, the coefficients increase significantly from 0.25 percentile to 0.50 percentile and then decrease significantly from 0.50 percentile to 0.75 percentile, confirming an inverted V-shaped relationship. The pattern for migrant workers differs: the coefficient from 0.25 percentile to 0.50 percentile has a *p*-value of 0.52, followed by a significant decrease from 0.50 percentile to 0.75 percentile. In contrast, educational level, productive agricultural machinery, durable products, cash gift, and out-of-pocket expenses for inpatient care exhibit a V-shaped relationship, with coefficients decreasing significantly from 0.25 percentile to 0.50 percentile and then increasing significantly from 0.50 percentile to 0.75 percentile.

**Table 5:** Wald test results for coefficient differences across quantiles of explanatory variables.

Explanatory variables	Joint Wald Test	Pairwise test: Q25 vs. Q50	Pairwise test: Q50 vs. Q75	Pattern
Educational level	17.00*	0.0942* (0.0164)	0.0533* (0.0164)	V-shaped
Health status	3.37*	0.0471* (0.0214)	0.0484* (0.0187)	Inverted V-shaped
Labor force	29.65*	0.0536* (0.0071)	0.0392* (0.0073)	Inverted V-shaped
Migrant workers	5.10*	0.0274 (0.0141)	0.0398* (0.0126)	Inverted V-shaped
Productive agricultural machinery	6.18*	0.0450* (0.0131)	0.0292* (0.0140)	V-shaped
Durable products	5.75*	0.0102* (0.0031)	0.0082* (0.0031)	V-shaped
Cash gift	28.03*	0.0171* (0.0038)	0.0199* (0.0027)	V-shaped
Inpatient	3.10*	0.1565* (0.0699)	0.1608* (0.0736)	Inverted V-shaped
Out-of-pocket expenses for inpatient care	5.28*	0.0247* (0.0082)	0.0230* (0.0089)	V-shaped

The joint Wald test reports an F-statistic. The pairwise test columns report coefficient differences, with standard errors in parentheses.

*p<0.05.

To test the underlying mechanism that larger labor force size and a higher number of migrant workers are associated with greater health poverty vulnerability, this paper divides the sample into farming and non-farming households based on whether the household engages in agricultural production, and separately estimates the effects of labor force size and the number of migrant workers on health poverty vulnerability. Table 6 reports the subgroup regression results.

To present the results for labor force size and the number of migrant workers clearly, the table reports only the coefficients for these two variables, although all explanatory variables were included in the analysis. In the farming household group, the coefficients for both labor force size and the number of migrant workers are significantly positive, indicating that increases in labor force/migrant work significantly elevate health poverty vulnerability in these households. In the non-farming household group, the coefficient for labor force size remains significantly positive, but the coefficient for the number of migrant workers is no longer significant. Tests of equality across groups show that the coefficient for the number of migrant workers differs significantly between the two groups at the 5% level. These results reveal that while the positive effect of labor force size is universal, the heterogeneous effect of migrant work provides key evidence for the core mechanism of health damage caused by physical labor. Migrant work significantly increases vulnerability only in farming households, but not in non-farming households, and the between-group difference is significant. This suggests that the impact of migrant work on vulnerability is highly dependent on the nature of labor. Migrant workers from farming households are more likely to engage in high-intensity physical labor, thereby accelerating health capital depletion. To further enhance the credibility of this conclusion, we also conducted a supplementary analysis of interaction effects. The interaction model shows that the interaction term between the number of migrant workers and farming households is significantly positive, further confirming that the impact of migrant work on vulnerability is stronger in farming households (see Table 7).

**Table 6:** Grouped regression results (farming vs. non-farming households).

Explanatory variables	Farming households (*N* = 3,206)	Non-farming households (*N* = 1,165)	Tests of equality across groups
Labor force	0.0576* (0.0053)	0.0593* (0.0092)	0.03
Migrant workers	0.0661* (0.0091)	0.0196 (0.0147)	6.63*

Standard errors in parentheses. The test of differences across groups reports the chi-squared value.

*p<0.05.

**Table 7:** Interaction effect regression results.

Explanatory variables	Model 1	Model 2
Labor force	0.0504* (0.0069)	0.0585* (0.0046)
Migrant workers	0.0536* (0.0077)	0.0195 (0.0136)
Farming household	−0.0517* (0.0199)	−0.0459* (0.0136)
Labor force × farming	0.0106 (0.0070)	—
Migrant workers × farming	—	0.0472* (0.0156)

Standard errors in parentheses.

*p<0.05.

#### 4.3.3 Robustness test

**Figure 2:**
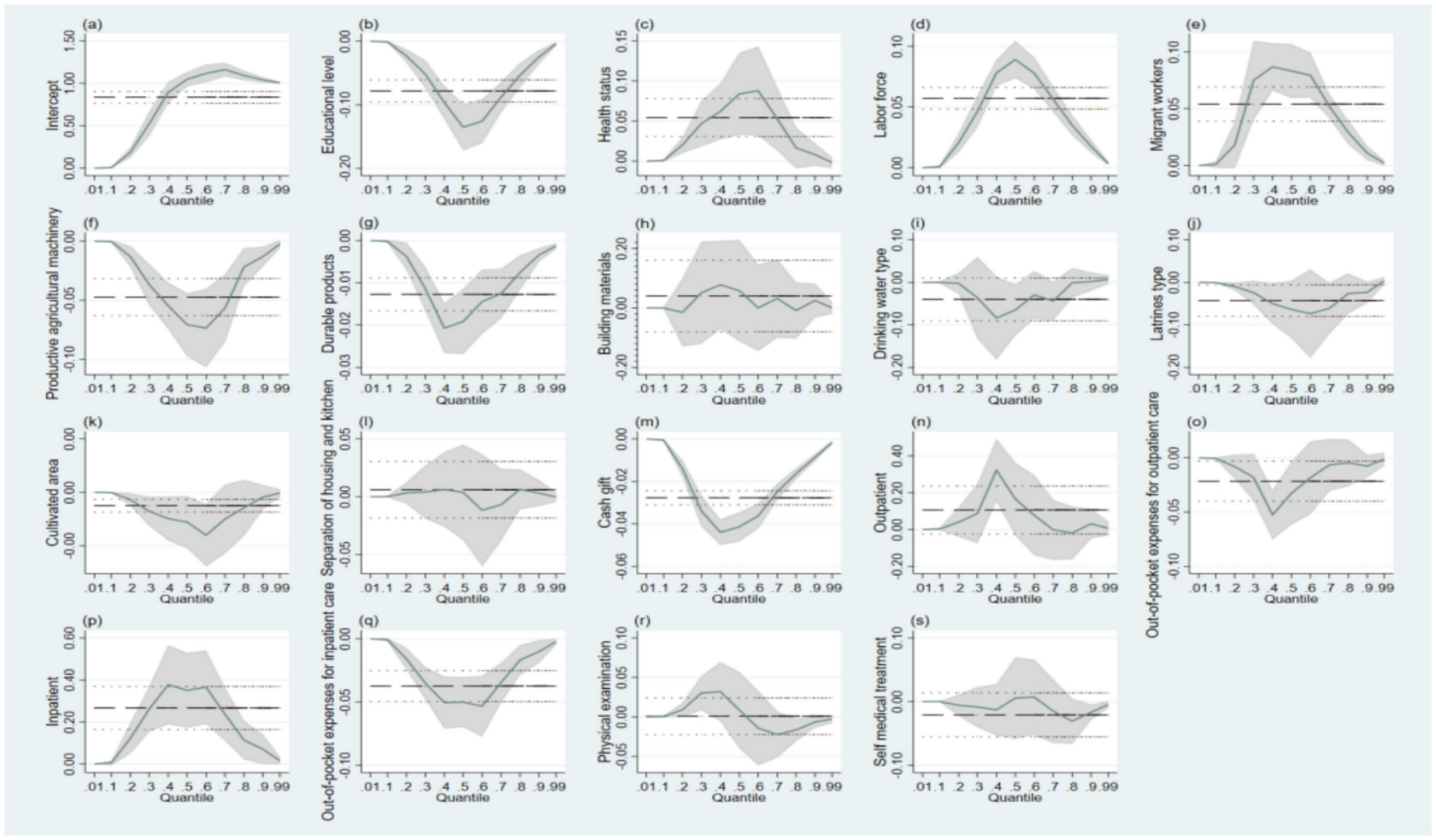
Full quantile regression results of resource endowment, medical services utilization and health poverty vulnerability of middle-aged and older households. **(a)–(s)** denote the specific variables of resource endowment, medical services utilization and health poverty vulnerability.

In order to verify the differences in the impact of resource endowment and medical services utilization on health poverty vulnerability at various quantiles, a full quantile regression model was further used to analyze the marginal contribution and trends of each independent variable across all quantiles. Taking the educational level in resource endowment and outpatient in medical services utilization as examples (Figure 2). Figure 2b reflects that as the percentile increases, the educational level of households initially declines and then rebounds, with the lowest point at the 0.50 percentile, forming a V-shaped relationship. The overall trend in Figure 2n shows an inverted V-shaped relationship. The full quantile regression is consistent with the results presented in Table 4, confirming the robustness of the main findings and estimation results.

### 4.4 Structural equation modeling analysis

#### 4.4.1 Model fit

**Figure 3:**
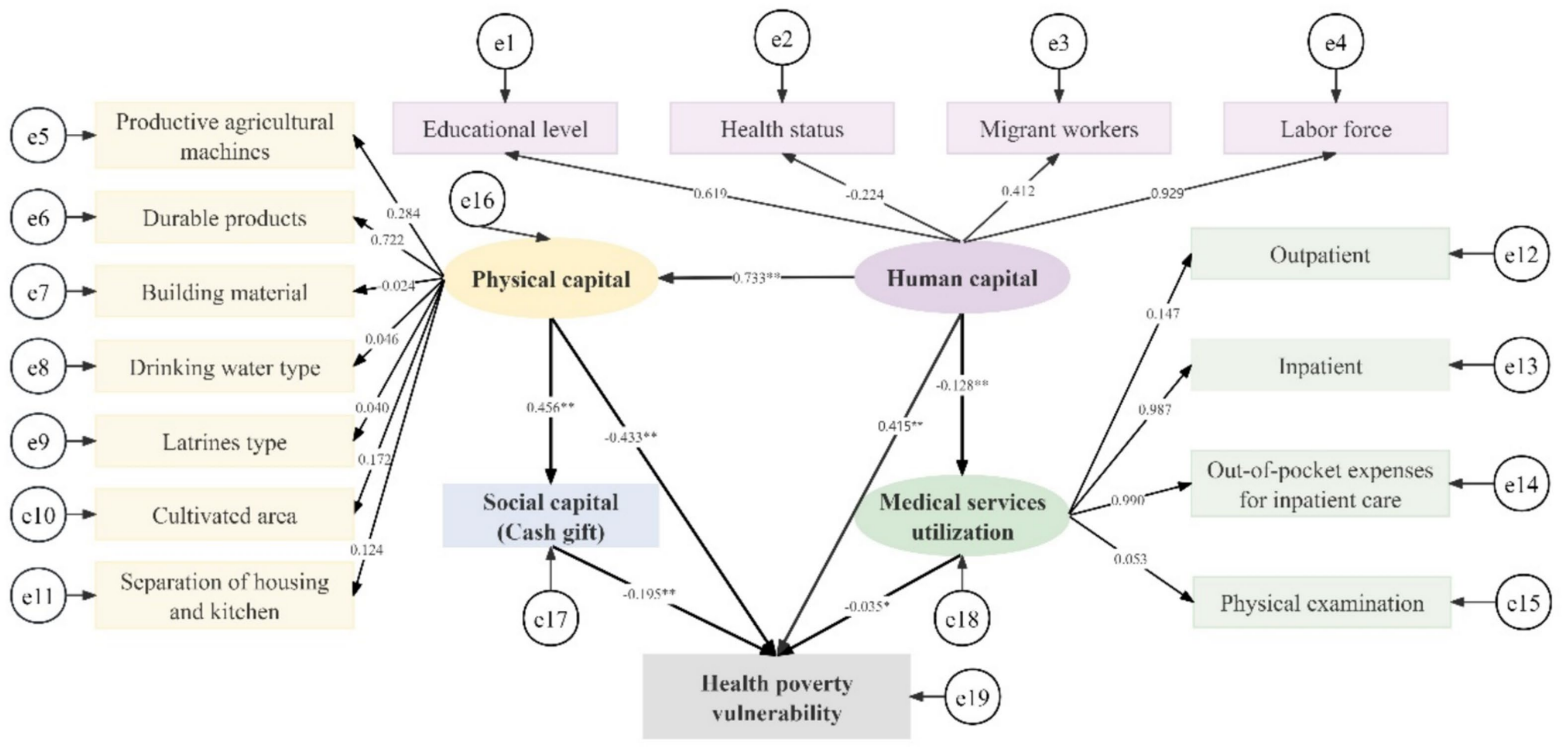
The modified model of resource endowment, medical services utilisation and health poverty vulnerability of middle-aged and older households in rural western China. *p<0.05, **p<0.001. The numbers above the “→” are standardized coefficients.

Amos software was used to draw model path diagrams and perform data operations. Running the initial model (Figure 2), the modification indices output from Amos were examined. The initial model fit indices are as follows: GFI = 0.883; AGFI = 0.848; PGFI = 0.683; IFI = 0.554; CFI = 0.554; SRMR = 0.556; RMSEA = 0.150, indicating poor model fit. The modification indices (MI) were checked in descending order. Starting from the largest modification indices value, each suggested modification was considered only if it was conceptually supported by the existing literature. Adjustments were made one at a time in a stepwise manner to avoid capitalizing on chance. During the model modification process, we screened the initial measurement indicators based on modification indices and theoretical judgment. The results showed that the modification indices for out-of-pocket expenses for outpatient care and physical examination were relatively high, and their measurement errors were significantly correlated with multiple indicators. From a theoretical perspective, out-of-pocket expenses for outpatient care are susceptible to recall bias, while physical examination tends to reflect preventive behavior rather than actual utilization intensity. Therefore, taking these factors into consideration, we removed these two indicators in the revised model. After deletion, the model fit indices improved substantially, and the remaining indicators effectively captured the intensity and frequency of health service utilization. The modified model is illustrated in Figure 3. Overall, model fit indices were satisfactory (GFI = 0.973 > 0.90; AGFI = 0.964 > 0.90; PGFI = 0.725 > 0.5; IFI = 0.956 > 0.90; CFI = 0.956 > 0.90; SRMR = 0.038 < 0.05; RMSEA = 0.042 < 0.05).

#### 4.4.2 Path analysis

The estimated path coefficients of the model are shown in Table 8, with all paths being statistically significant. Among the pathways affecting the health poverty vulnerability of rural middle-aged and older households, the path coefficient of physical capital was the largest, followed by human capital. Household physical capital is significantly negatively correlated with health poverty vulnerability, showing a potential buffering effect, which may help alleviate the risk of illness-induced poverty for rural middle-aged and older households in the study area. Notably, the effect of human capital on health poverty vulnerability was positive. Medical service utilization is significantly negatively correlated with health poverty vulnerability, indicating its potential positive role in alleviating health poverty vulnerability in the study context. For resource endowment, human capital positively influenced physical capital, which in turn positively affected social capital. Human capital was the only factor that influenced medical services utilization. Table 9 shows the results for all direct, indirect and total effects. Human capital and physical capital had direct and indirect effects on health poverty vulnerability, while the remaining pathways had only direct effects.

**Table 8:** Results of path coefficient estimation.

Path	Standardized path coefficient	S.E.	C.R.	*p*-value
Human capital→physical capital	0.733	0.021	14.689	<0.001
Human capital→medical services utilization	−0.128	0.017	−7.862	<0.001
Human capital→health poverty vulnerability	0.415	0.033	10.382	<0.001
Physical capital→social capital	0.456	0.565	14.060	<0.001
Physical capital→health poverty vulnerability	−0.433	0.106	−7.961	<0.001
Social capital→health poverty vulnerability	−0.195	0.002	−10.589	<0.001
Medical services utilization→health poverty vulnerability	−0.035	0.012	−2.421	<0.05

**Table 9:** Results of standardized effects decomposition.

Path	Direct effects	Indirect effects	Total effects
Human capital→physical capital	0.733	—	0.733
Human capital→medical services utilization	−0.128	—	−0.128
Human capital→health poverty vulnerability	0.415	−0.379	0.036
Physical capital→social capital	0.456	—	0.456
Physical capital→health poverty vulnerability	−0.433	−0.089	−0.522
Social capital→health poverty vulnerability	−0.195	—	−0.195
Medical services utilization→health poverty vulnerability	−0.035	—	−0.035

## 5 Discussion

In rural areas of western China, we investigated the effects of resource endowment and medical services utilization on households’ health poverty vulnerability at different quantiles. We used structural equation modeling to provide a comprehensive understanding of the pathways linking resource endowment, medical services utilization, and health poverty vulnerability. Our finding that medical utilization reduces vulnerability diverges from literature on “medical impoverishment.” This is likely due to Ningxia’s “First-treat, Post-paid” policy and the triple-tier medical insurance system, which significantly lowers the immediate out-of-pocket burden. This buffering effect ensures that healthcare seeking prevents minor illnesses from escalating into catastrophic events. For households with “moderate vulnerability,” we propose targeted “Asset-Building Programs,” such as machinery cooperatives, to leverage the high sensitivity of this group to physical capital improvements.

### 5.1 Rural middle-aged and older households of Ningxia have high health poverty vulnerability

This study utilized survey data from rural middle-aged and older households of Ningxia in 2022, applying the poverty line at 60% of per capita disposable income and the vulnerability criterion of 0.5 to measure the health poverty vulnerability. The result shows that the vulnerability of these households is 31.6% higher than that of the full sample of local households (26). The 48% overall health poverty vulnerability rate accurately reflects the severe realistic situation of middle-aged and older rural households in southern Ningxia, western China. This higher vulnerability can be attributed to the unique physiological characteristics, disease patterns, and socio-economic status of middle-aged and older individuals, as well as the acceleration of empty nesting and aging trends in rural households (41, 42). These factors make them more prone to falling into health poverty and increasing vulnerability in the future. Furthermore, the health poverty vulnerability observed in Ningxia is not significantly different from that of rural older populations in Hubei and Guizhou provinces (43). However, the relatively high vulnerability in Ningxia is uniquely tied to its rural industrial structure, which is dominated by high-intensity manual farming that accelerates physical “wear and tear.” Unlike Hubei, where labor mobility is higher, Ningxia’s middle-aged and older population remains more “anchored” to the land, leading to a specific chronic disease spectrum (e.g., respiratory and musculoskeletal disorders) that requires long-term, low-intensity medical resource allocation (44, 45). This reinforces the need to address the health poverty vulnerability of rural middle-aged and older households. It is also crucial to identify and address the factors affecting these households, with particular attention to those with moderate health poverty vulnerability, who are most affected.

### 5.2 The impact of resource endowment and medical services utilization on health poverty vulnerability of quantile regression

In this study, quantile regression analysis was used to examine the impact of resource endowment and medical services utilization on health poverty vulnerability. The results from the quantile regression model revealed that the coefficients of health status, labor force, migrant workers, and inpatient care utilization were positive and significant across households with different quantiles of health poverty vulnerability. This indicates that, due to the specific physical characteristics, disease patterns, and the geographical and economic constraints faced by middle-aged and older people, households affected by chronic diseases are particularly vulnerable to health-related risks and poverty (44). Therefore, older households with chronic diseases in rural areas are particularly vulnerable to health poverty (43). Consistent with the findings of this study, middle-aged and older households with chronic diseases and those utilizing inpatient services face higher health poverty vulnerability. Contrary to our expectations, the analysis revealed that households with a larger labor force and more migrant workers had higher health poverty vulnerability. This may be due to the nature of rural economies, where the labor force and migrant workers are often the primary income sources. On one hand, as in the analysis of subgroup regression and interaction effects in this study. The physical labor associated with their work may impair health over time, leading to increased medical costs and reduced economic output. Migrant workers from farming households are more likely to engage in high-intensity physical labor, thereby accelerating health capital depletion. On the other hand, other family members may experience a heavy psychological burden from caring for a sick household member, which could further reduce their productivity and exacerbate their overall vulnerability to health poverty (46, 47). The path coefficient (0.456) underscores that physical assets serve as the “entry ticket” to rural social networks (Renqing). However, we observed a high standard error (0.565) in this estimation, reflecting the high heterogeneity of social reciprocity in Ningxia. In some villages, social capital is driven more by clan prestige than by modern physical wealth, suggesting that the “wealth-to-social status” conversion is not uniform across the region. Despite this variance, the significance (*p* < 0.001) confirms that asset-building is a viable indirect strategy for risk mitigation.

Educational level, household productive agricultural machines, durable products, cash gift, and inpatient own expense all have statistically significant negative effects on health poverty vulnerability. In other words, these indicators can significantly reduce the vulnerability of households to health-related poverty. To combat poverty caused by illness, in addition to health poverty alleviation policy, Targeted Poverty Alleviation Project also include policies such as education poverty alleviation. Our findings align with previous studies, suggesting that improvements in education increase a household’s ability to generate income and manage expenses, making it less likely for them to be impoverished (48, 49). This demonstrates that education and health poverty alleviation policies are mutually reinforcing (50). Furthermore, the negative effect of educational level is the most significant in this study, revealing the effectiveness and importance of education in alleviating health poverty. The relevant study has shown that the utilization of productive agricultural machines is a pivotal way to eradicate poverty among farmers (51). This study found that an increase in the number of productive agricultural machines can reduce the health poverty vulnerability of households. One possible reason is that mechanized farming reduces the demand for strenuous physical labor, improving both work quality and life quality, while minimizing health risks. Additionally, productive agricultural machinery enhances productivity and efficiency, thereby reducing the need for migrant labor (52). As highlighted in the previous section on labor force and migrant workers, this situation can be improved by increasing the use of productive agricultural machines. Building on previous findings, we identified that household durable products form the core of household materials, with their quantity and quality directly tied to the living standards and needs of residents, which can measure social well-being (53, 54). This helps explain why the increase in the number of durable products is associated with a reduction in households’ health poverty vulnerability, as observed in this study. Additionally, gift exchange and gifting play a significant role in social interaction. We found that as cash gifts increase, health poverty vulnerability decreases. Although the impact was relatively small, it still indicated that rural residents in Ningxia recognize the importance of human connection. Social capital improves household resilience to risk. Its economic cooperation and civil mutual assistance functions helping to mitigate health risk shocks. The coverage of health insurance has a significant role in reducing the risk of CHE. This study found that the higher inpatient out-of-pocket expenses for middle-aged and older households in rural Ningxia, the lower risk of health poverty. This probably due to the benefits of health insurance coverage and the supporting policies and systems.

### 5.3 The pathways of resource endowment, medical services utilization, and health poverty vulnerability

This study used structural equation modeling to analyze the pathways of resource endowment, medical services utilization, and health poverty vulnerability. This contradicts some literature on poverty caused by illness, likely because our sample period coincides with the enhanced implementation of the “Basic Medical Insurance + Serious Illness Insurance + Medical Assistance” triple-tier protection in Ningxia. This policy framework effectively shifts the impact of utilization from wealth drain to health investment. Among all the paths, human capital had the greatest effect on physical capital. The study suggests that human capital is the foundation for farmers to develop various livelihood activities (17). Our findings support this view, indicating that human capital is relatively important in rural households in Ningxia, and its quantity and quality determine the use of physical capital. Furthermore, human capital shows a direct positive effect on vulnerability. The path results related to health poverty vulnerability show that increasing the stocks of human capital in resource endowment tends to increase the health poverty vulnerability of rural households. This is due to the latent variable’s heavy loading on “chronic illness,” which acts as a risk factor. This finding aligns with the results from the quantile regression, where health status, labor force and migrant workers all had positive effects on vulnerability. This suggests a suppression effect where the negative influence of education is outweighed by the positive influence of health impairment within the overall human capital construct.

The path analysis of health poverty vulnerability showed that increasing the stocks of physical and social capital in resource endowment reduced vulnerability. Among these, physical capital plays a particularly significant role. This may be because physical capital can be effectively expanded in a short period compared to human and social capital, allowing for rapid improvements in the capital status of households (55). The utilization of medical services by households reduces health poverty vulnerability. This result contradicts the findings of earlier studies that suggest the higher utilization rate of health services in the western region leads to impoverishment due to medical expenses (56). One possible explanation for this result is that welfare policies have effectively increased the ability of rural households to withstand the economic risks associated with illness (3). For instance, the reimbursement policy in the medical insurance system and the ‘First-treat Post-paid’ pattern may have alleviated financial burdens. Additionally, the improvement in health literacy among residents could be a contributing factor. In the context of high disease incidence in the middle-aged and older population, residents may be more inclined to seek medical treatment when their conditions are manageable, thus preventing higher medical costs from delaying optimal treatment. Furthermore, the path coefficient of medical services utilization is −0.035 (*p* < 0.05, C.R. = − 2.421). Although weak, it indicates a significant buffering role, and the modest effect size may stem from self-reporting bias in rural surveys. Based on the “sensitivity peak” identified in our quantile regression, policy interventions should prioritize households with moderate vulnerability (index 0.25–0.75). For this group, marginal improvements in physical capital yield the highest reduction in poverty risk. Specifically, the “core path of physical capital” should be activated through agricultural machinery cooperatives. This would allow households to pool physical assets, thereby building social capital (collective connection) while reducing individual physical labor intensity. Finally, given the weak but significant effect of medical utilization (−0.035), policy should shift from simply “increasing access” to “enhancing financial protection.” Efforts should focus on increasing the reimbursement ratio for chronic disease outpatient services and expanding the scope of “one-stop” settlement services to minimize out-of-pocket ratios, which is the primary driver of vulnerability in the 45 + age group (57).

## 6 Limitaions of the study and future research directions

This section discusses the limitations of this study, all directly linked to our core empirical findings and research design, and proposes targeted future research directions.

First, the generalizability of our findings is strictly limited by the sample scope, and cannot be inappropriately extended beyond Ningxia. Our sample covers 4,371 middle-aged and older rural households in southern Ningxia, an underdeveloped western region with unique medical institutional and demographic characteristics. The findings can only be generalized to western rural areas with similar economic and institutional contexts, and cannot be extended to urban households, developed eastern rural areas, or non-older groups.

Second, our cross-sectional study design has inherent limitations for causal inference, which we critically discuss in relation to our research claims. Single-time-point survey data can only capture statistical correlations between variables, but cannot establish strict temporal precedence or rule out reverse causality. Our causal path hypotheses are based on classic theoretical frameworks, but empirical results cannot provide definitive causal evidence, requiring extreme caution for relevant causal claims.

Third, the study has key inherent methodological limitations, including potential endogeneity from omitted unobservable factors and reverse causality, measurement bias from self-reported questionnaire data, and potential SEM overfitting risk from sample-specific model adjustment, even with our strict theory-driven modification rules.

Finally, all future research directions are fully aligned with the above limitations, including expanding sample scope, adopting panel data for causal inference, and optimizing variable measurement and model cross-validation.

## 7 Conclusion

The health poverty vulnerability of middle-aged and older rural households in rural areas of Ningxia is a comprehensive outcome influenced by various factors. This study presents a model that demonstrates the different impacts and pathways through which various resource endowments and medical services utilization affect the health poverty vulnerability of rural households. The results indicate that the health poverty vulnerability among middle-aged and older households in rural areas of western China is relatively high and warrants further attention. The impact of various influencing factors on households with moderate health poverty vulnerability is relatively greater compared to those with low or high levels, with education level having the most significant impact. Among the influence pathways, physical capital, because of its realizable characteristics, its stock is crucial in reducing health poverty vulnerability. Additionally, the utilization of medical services for middle-aged and older rural households in rural areas of western China emerges as another key factor in improving health poverty vulnerability.

This model can serve as a basis for developing targeted intervention measures. For middle-aged and older rural households in southern Ningxia, the study area, targeted policy recommendations are proposed based on our empirical findings. Firstly, rural development managers should focus on middle-aged and older households over the long term, assess their health poverty vulnerability, and establish dynamic monitoring systems to identify vulnerable groups. By doing so, they can consolidate and expand the achievements in poverty alleviation efforts. Secondly, efforts should be made to actively enhance the human capital and physical capital of middle-aged and older households, particularly those with moderate health poverty vulnerability, to prevent them from progressing to a higher vulnerability level. This can be achieved by offering professional skills training for adults with lower education levels to improve their labor skills and cultivate new types of farmers. Simultaneously, rather than simply restricting labor mobility, policies should focus on optimizing labor mobility patterns to reduce health risks associated with high-intensity migrant work. For instance, expanding subsidies for productive agricultural machinery can enhance local agricultural productivity, enabling laborers to reduce reliance on heavy physical labor through mechanization while maintaining stable income without leaving their hometowns. For physical capital, expanding subsidies for productive agricultural machines and other essential resources can enhance labor efficiency and productivity, as well as reduce the health risks associated with heavy physical labor. Finally, it is crucial to strengthen the promotion of health knowledge, raise awareness of prevention, and encourage timely medical treatment. Controlling medical service costs and further integrating the health insurance system will help increase the resilience of rural residents in western China to the economic risks of illness.

## Data Availability

The data have been licensed for use in the current research. Access to the data can be obtained from the corresponding author upon reasonable request.
